# A systematic review of how missing data are handled and reported in multi‐database pharmacoepidemiologic studies

**DOI:** 10.1002/pds.5245

**Published:** 2021-05-07

**Authors:** Nicholas B. Hunt, Helga Gardarsdottir, Marloes T. Bazelier, Olaf H. Klungel, Romin Pajouheshnia

**Affiliations:** ^1^ Division of Pharmacoepidemiology and Clinical Pharmacology, Utrecht Institute for Pharmaceutical Sciences (UIPS) Utrecht University Utrecht The Netherlands; ^2^ Department of Clinical Pharmacy University Medical Centre Utrecht Utrecht The Netherlands; ^3^ Department of Pharmaceutical Sciences, School of Health Sciences University of Iceland Reykjavik Iceland

**Keywords:** missing data, multi‐database, pharmacoepidemiology, review

## Abstract

**Purpose:**

Pharmacoepidemiologic multi‐database studies (MDBS) provide opportunities to better evaluate the safety and effectiveness of medicines. However, the issue of missing data is often exacerbated in MDBS, potentially resulting in bias and precision loss. We sought to measure how missing data are being recorded and addressed in pharmacoepidemiologic MDBS.

**Methods:**

We conducted a systematic literature search in PubMed for pharmacoepidemiologic MDBS published between 1st January 2018 and 31st December 2019. Included studies were those that used ≥2 distinct databases to assess the same safety/effectiveness outcome associated with a drug exposure. Outcome variables extracted from the studies included strategies to execute a MDBS, reporting of missing data (type, bias evaluation) and the methods used to account for missing data.

**Results:**

Two thousand seven hundred and twenty‐six articles were identified, and 62 studies were included: using data from either North America (56%), Europe (31%), multiple regions (11%) or East‐Asia (2%). Thirty‐five (56%) articles reported missing data: 11 of these studies reported that this could have introduced bias and 19 studies reported a method to address missing data. Thirteen (68%) carried out a complete case analysis, 2 (11%) applied multiple imputation, 2 (11%) used both methods, 1 (5%) used mean imputation and 1 (5%) substituted information from a similar variable.

**Conclusions:**

Just over half of the recent pharmacoepidemiologic MDBS reported missing data and two‐thirds of these studies reported how they accounted for it. We should increase our vigilance for database completeness in MDBS by reporting and addressing the missing data that could introduce bias.

**Key Points**

- Missing data can lead to biased estimates of drug safety and effectiveness, a problem that can be exacerbated in multi‐database studies.
- Forty‐four percent of recent multi‐database pharmacoepidemiologic studies did not report missing data and 69% did not report accounting for missing data.
- In studies which report missing data, lifestyle variables were most frequently reported missing (14%–29%).
- Most studies which reported a method to address missing data performed a complete case analysis (68%). Multiple imputation was the predominant statistical method used to handle missing data (22%).
- Variables with missing data, the potential bias and accounting for missing data should be thoroughly reported.

## 1 INTRODUCTION

The use of multiple health databases in pharmacoepidemiologic studies can facilitate more robust assessments of drug safety and effectiveness.^1^, ^2^ Multi‐database studies (MDBS) involve the analysis of routinely collected data from two or more data sources, which may take the form of health insurance claims databases, electronic healthcare records (EHR) or healthcare record linkage systems.^3^ MDBS can allow us to investigate specific subgroups of patients, rare outcomes, or conduct an early post‐approval assessment of safety and effectiveness.^4^, ^5^ Multi‐national MDBS can lead to more generalisable results, due to the inclusion of heterogeneous patient populations^1^ and allow us to compare the safety and effectiveness of compounds between countries and regions, taking differences in ethnology and health care systems into account.^5^

MDBS are methodologically more complex than single database studies as a result of inter‐database heterogeneity, caused by differences in what information is recorded and how. This brings practical challenges such as how to standardise analyses across a distributed network and how to combine data or results.^2^, ^6^, ^7^ In addition, there may also be differences in the completeness of information across databases, potentially resulting in missing data, herein defined as any data that are relevant to the analysis or interpretation of a study that are not available to the study investigators at the time of analysis or reporting. Common data models (CDMs) and common protocols (CPs) have been used across database networks to mitigate bias due to heterogeneity in MDBS analyses,^2^ but cannot solve differences in database completeness. Failure to account for missing data in epidemiologic studies can introduce bias, even altering the direction of treatment effect estimates, and reduce the precision of effect estimates.^8^, ^9^ For example, one study showed that risk estimates of venous thromboembolism associated with anti‐osteoporotic medications were substantially affected by the use of different strategies for the handling of missing data, leading to differences in the direction of treatment effect estimates.^8^

Missing data can arise at several stages within a multi‐database pharmacoepidemiologic study. Like in a single database study, data may not be recorded at the stage of data entry into the database. For example, data may be partially (or sporadically) missing because a health professional recorded information in an unstructured manner (e.g., using free text) or did not collect information for certain patients, such as body mass index (BMI) and smoking status, because they were considered healthy.^10^, ^11^, ^12^ MDBS can have the additional complexity of completely (or systematically) missing variables, where data on a certain variable are missing for all individuals in a database.^12^, ^13^ This may occur when certain variables are not recorded in a database because they are not required by the health authorities for reimbursement (in administrative/claims databases) or because the recording is not part of routine clinical practice (in electronic health record (EHR) databases). In the case of a systematically missing confounder, this can lead to residual confounding in a study; if a variable used to define exposure is systematically missing, this can lead to exposure misclassification in one or more of the databases. There may also be some information loss during the extract, transform and load process (e.g., data which did not meet the quality criteria are omitted) or creation of final analytical variables. This can happen, for example, when components of a composite variable are missing or if time restrictions are applied to a variable, such as a measurement being available within 12 months of the index date.^14^

Methods to account for sporadically missing data, such as multiple imputation (MI) and inverse probability weighting, are widely known.^8^, ^15^ To handle systematically missing data, a practical approach is to exclude the missing variable from the analyses or exclude an entire database.^8^ A recently proposed alternative is multi‐level MI (MLMI), which can account for both sporadically and systematically missing data. This approach utilises information on the covariance of variables in one dataset or database to impute missing information in another.^16^, ^17^, ^18^, ^19^

The reporting of missing data and the methods used to address it are specified in the RECORD‐PE and STROBE statements.^20^, ^21^ Without thorough reporting, we cannot have full confidence in the validity of the study estimates. Our aim for this review was to first measure the extent to which recent MDBS reported missing data and considered it as a potential source of bias. Second, we sought to identify which strategies are being reported in MDBS to deal with missing data.

## 2 METHODS

### 2.1 Study design and search criteria

We conducted a systematic literature search in PubMed to identify and report the methods used in recent peer‐reviewed, multi‐database pharmacoepidemiologic studies. The full list of inclusion and exclusion criteria can be seen in Table 1. The search strategy was adapted from a previous review of MDBS by Bazelier et al.^1^ (see supplementary table 1 for the PubMed search terms). We restricted the search to studies published between 01‐01‐2018 and 31‐12‐2019. This search was performed on 07‐11‐2019 for the studies up until 31‐10‐2019. The search was updated on 08‐01‐2020 to include the studies between 01‐11‐2019 and 31‐12‐2019.

**TABLE 1:** The selection criteria for published pharmacoepidemiologic multi‐database studies to be included in the systematic review

Inclusion criteria	Exclusion criteria
Multi‐database (of independent patient populations)	Spontaneous reporting databases
Observational (non‐randomised) study	Methodological studies
Safety or effectiveness of a pharmaceutical compound	Drug utilisation studies
Publish date 01‐01‐2018 to 31‐12‐2019	Reviews
Peer‐reviewed literature	Clinical trials
	Literature which is not published in English
	Non‐routine data collection where data were collected for research purposes

We additionally performed a search of established database networks. These networks were identified by the authors, with the assistance of an external expert. The networks included those published by the European Network of Centres for Pharmacoepidemiology and Pharmacovigilance (ENCePP), the US Food and Drug Administration's (FDA) Sentinel Initiative, the Canadian Network for Observational Drug Effect Studies (CNODES), the Asian Pharmacoepidemiology Network (AsPEN), the National Patient‐Centered Clinical Research Network (PCORnet) and the Vaccine Safety Datalink (VSD).^22^, ^23^, ^24^, ^25^, ^26^, ^27^ Where the network websites were not up‐to‐date with a full list of associated publications, the names of the networks were searched in PubMed (23‐01‐2020).

### 2.2 Screening and selection

Title and abstract screening for eligibility were performed by one author (NBH). Another author (RP) independently screened the title and abstract of 100 articles. Any differences in this pilot screening were discussed and resolved. One author (NBH) then screened the full‐text of the remaining articles for eligibility. Two authors (NBH and RP) independently reviewed the full list of included studies to confirm eligibility and disagreements were discussed with the other authors.

### 2.3 Extraction of the general study characteristics

The general characteristics of each study were recorded: The study design (e.g., cohort or case–control), the journal, the exposure and outcome, the size (number of databases, countries, subjects), the database type (administrative/claims, EHR, other) and whether the study provided a pooled estimate. To categorise the strategies used to execute the MDBS, we used criteria described by ENCePP (a full description can be found in Gini et al.^28^). We categorised each study as carrying out either a local analysis, where the data extraction and analysis are conducted by individual centres (according to a CP); sharing of raw data, where the local site extracts the raw data and transfers it to a central partner for the analysis; the use of a study‐specific CDM; or use of a general CDM.^28^

### 2.4 Extraction of the outcomes

For the primary outcome, we measured how many of the included studies reported the existence of missing data; in what context this was reported (methods, results or as a limitation); which variables were missing data; type of missingness (sporadic or systematic, as determined by the authors); the type of variable with missing data (exposure, outcomes or confounders); and the amount of % missing. We recorded whether the authors of each study discussed the extent to which the missing data had contributed to bias and whether missing data were missing completely at random (MCAR), missing at random (MAR) or missing not at random (MNAR).^29^

For studies that reported missing data, we recorded which method was used to account for missing data. For studies which used MI, we additionally extracted information on the number of imputations per variable, the number of imputed variables and the statistical software used for the imputation.

### 2.5 Data analysis

Data extraction was piloted in five articles by two authors (NBH and RP). The remaining data extraction was conducted by a single author (NBH) and any uncertainties were discussed with the co‐authors. Extracted data were recorded and where applicable, data were transformed into a pre‐specified answer list. Means, medians and ranges were calculated.

## 3 RESULTS

**FIGURE 1:**
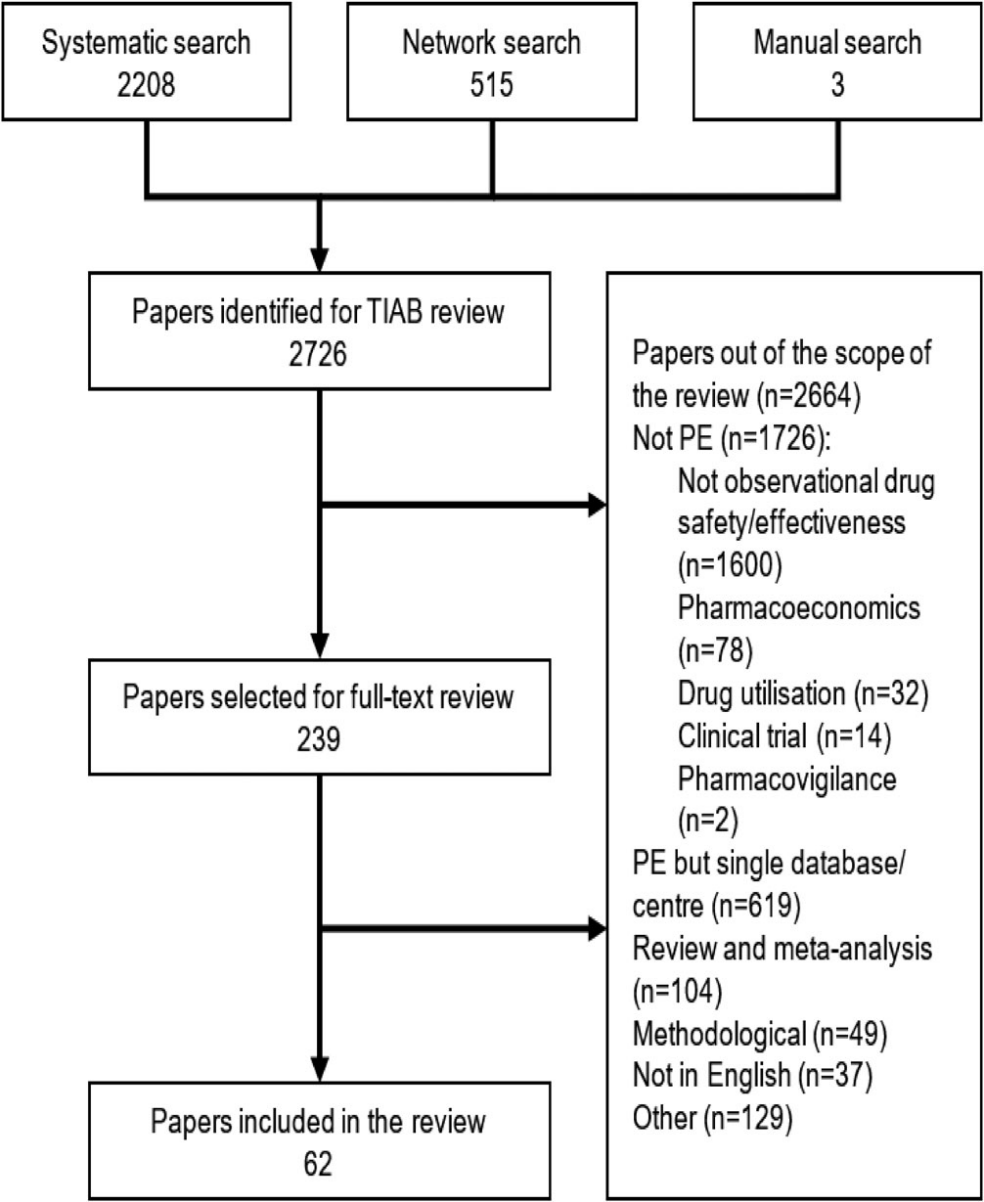
A flow diagram showing the selection process for the papers included and excluded from this systematic review. Other (*n* = 129): Presentations (62), case reports (29), not in human studies (8), letter/ comments (8), full‐text unavailable (7), guidelines (4), duplicate (4), protocols (2), conference abstracts (1), book chapters (1), not in timeframe (1), and strategic plans (1). TAIB, title or abstract; PE, pharmacoepidemiology

The search identified 2726 publications for title/abstract review (Figure 1). Sixty‐two articles from forty‐four scientific journals were eligible for inclusion (see supplementary table 2 for a list of included articles). An overview of the general study characteristics can be seen in Table 2. Thirty‐five (56%) of the included studies used exclusively North American data, 19 (31%) used exclusively European data, 7 (11%) used data from a combination of regions and 1 (2%) used exclusively East‐Asian data (Table 2 and supplementary figure 1).

**TABLE 2:** General characteristics of the included multi‐database pharmacoepidemiologic studies

Study characteristics		
*Safety or effectiveness*	*N (62)*	*%*
Safety	50	80
Effectiveness	6	10
Both	6	10
*Study design*	*N (62)*	*%*
Cohort	53	85
Case–control	9	15
*Exposure ATC anatomical group*	*N (62)*	*%*
Anti‐infectives for systemic use	17	27
Alimentary tract and metabolism	10	16
Antineoplastic and immunomodulating agents	8	13
Cardiovascular system	6	10
Blood and blood‐forming organs	6	10
Nervous system	5	8
Genitourinary system and sex hormones	2	3
Multiple groups	5	8
Other	3	5
*Database type*	*N (62)*	*%*
Administrative/claims	28	45
Electronic health records	12	19
Other	4	6
Combination	18	29
*Study size (individuals)*	*Mean*	*Range*
Cohort	324 190	568–6 177 795
Case–cohort	363 567	193–1 706 113
No. of databases	5	2–17
No. of countries	2	1–9
MDBS strategies		
*Pooled estimate*	*N (62)*	*%*
Yes	57	92
No	5	8
*MDBS strategy (ENCePP criteria)*	*N (62)*	*%*
Sharing of raw data	10	16
General CDM	23	37
Study‐specific CDM	1	2
Local analysis	22	35
Not reported	6	10

Abbreviations: ATC, anatomical therapeutic chemical; MDBS, multi‐database study; CDM, common data model; ENCePP, European Network of Centres for Pharmacoepidemiology and Pharmacovigilance.

Fifty‐seven (92%) of the included studies provided a pooled estimate of multiple databases in their analysis. Twenty‐two (35%) of the included studies carried out their analysis locally per study site, of which five did not provide a pooled estimate of multiple databases. Ten (16%) reported sharing raw data for a central analysis and 24 (38%) used a CDM. Twenty‐three (96%) of these used a general CDM with 11 using the VSD and 7 using Sentinel's CDM; one study used a study‐specific CDM.

Missing data were reported in 35 (56%) articles, with potential confounders such as lifestyle factors reported missing most often: BMI (*n* = 10, 29%), smoking status (*n* = 9, 26%), age or date of birth (*n* = 7, 20%), ethnicity/race (*n* = 6, 17%) and education level (*n* = 5, 14%). The extent of the reporting varied greatly and in six cases missing data were inferred from close inspection of the text or tables. Five articles reported the percentage of missing data, either by reporting the percentage per variable or per individuals in a database with data missing on one or more variable. The amount of missing data (per variable or database) reported in studies ranged from <1% to 56%. For the other 27 (44%) studies, no evidence of missing data was reported. In those that used a CDM (*n* = 24), 58% reported missing data compared to 55% of studies which did not use a CDM (*n* = 38). Missing data were less often reported in studies which used North‐American (54%) and European data (53%) compared to those which used data from a combination of regions (86%). The reporting of missing data differed between studies which used EHR data (67%), admin/claims databases (54%), a combination of EHR and admin/claims data (50%) and other types of databases such as registries (75%).

In the 35 studies which reported missing data, 13 (37%) reported the presence of sporadically missing data only, 4 (11%) reported the presence of systematically missing data only, 11 (31%) reported the presence of both and 7 (20%) were uncertain. Nine (26%) reported missing data in both the results and limitations sections, the rest reported in either section or not at all (Table 3). Fifteen articles (43%) reported that missing data existed within the constituent data sets without stating the quantity, while 14 (40%) reported the quantity of missing data as a percentage or number. Eleven (31%) studies reported that this missing data could have introduced bias to their findings, but no studies reported the possible missingness mechanism in terms of MCAR, MAR or MNAR.

**TABLE 3:** An overview the reporting of missing data and missing data methods in the 62 recently published multi‐database pharmacoepidemiology studies included in the systematic review

Reported missing data	*N* (62)	%
Yes	35	56
*Location*:		
In analysis	22	
In limitations	4	
In both	9	
*Variable type*:		
Exposure	5	
Outcome	8	
Confounder	31	
No	27	44
Evaluated missing data for bias		
Yes	11	18
No	51	82
Type of missingness		
Sporadic only	13	21
Systematic only	4	6
Both reported	11	18
Both uncertain	34	55
Types of missing variables reported		
Categorical only	11	18
Continuous only	6	10
Both	13	21
N/A or uncertain	32	52
Missing data method reported		
Yes	19	31
No	43	69
Missing data method used		
Complete case analysis (CCA)	13	21
Multiple imputation (MI)	2	3
Mean imputation	1	2
Substitution	1	2
Both MI and CCA	2	3
None or uncertain	43	69

In the 19 studies that reported a method to address missing, 13 (68%) carried out a complete case analysis, 2 (11%) used MI separately per database, 2 (11%) applied and compared a complete case analysis and MI, 1 (5%) used mean imputation and 1 (5%) substituted the missing information with information from a similar variable, where missing data points are replaced with the ‘best alternative’. For example, gestational age was determined by the first day of the last menstrual period if an ultrasound examination was missing. The studies which used both CCA and MI did so to test whether the separate strategies altered the overall outcome. For those that carried out MI, two studies reported the use of the Markov chain Monte Carlo method and two used MI by chained equations (MICE). Within one study which used MI, single imputation of the mode of the variable was used to address variables with <2% missing data. Studies imputed six (*n* = 2), four (*n* = 1), two (*n* = 1) or an unreported (*n* = 1) number of variables. The number of imputations per variable in the included studies which used MI were 20 (*n* = 2), 10 (*n* = 2) or unreported (*n* = 1). These imputations were performed in statistical software Stata (*n* = 2), R (*n* = 1), SAS (*n* = 1) or unreported (*n* = 1).

## 4 DISCUSSION

In this systematic review of recently published multi‐database pharmacoepidemiologic studies, we found that out of 62 included articles, only 56% reported missing data, 18% reported whether missing data could have biased the study and 31% reported how they dealt with missing data. The reporting of missing data was slightly higher in studies which used a CDM and those which used European and Asian data compared to North‐American data.

In contrast to Rioux et al.,^30^ which focused primarily on survey‐based observational research, instead of MDBS, we found a substantially lower proportion of studies reported missing data (56% compared to 87%), reported a method to address missing data (31% compared to 77%) and the possible mechanisms behind the missingness pattern (0% compared to 11%). However, the comparison with survey‐based observational research is not straightforward as we focus exclusively on database studies.

The inconsistent reporting of information about missing data observed in this review may also reflect that the completeness of information in healthcare databases varies according to the type of database and the type of information captured. EHR and health administrative databases are, by design, not created for the post‐marketing assessment of medicines, thus desired information for research, particularly on potential confounders, may not be recorded.^3^ This may be in contrast to registries where information that is not routinely collected in clinical practice is gathered through forms or surveys. In databases where there are certain elements which could be considered complete, such as information on pharmacy dispensing in databases used for reimbursement purposes; there still could be completely missing variables, such as lifestyle factors.

In MDBS which use claims databases, the issue of systematically missing data may be larger than that of sporadically missing data. In a pharmacoepidemiologic MDBS, there is a high likelihood of missing data because the multiple databases involved may record different variables. Furthermore, a recent study of established European health databases showed that vaccinations are captured in 38% of databases, while inpatient administered (5.8%) and over‐the‐counter drugs are rarely captured.^3^ It is therefore possible that the studies in this review have underreported missing data in their analyses, although this depends on the relevance of these kinds of exposures to the included studies.

The reporting of whether missing data could have contributed to potential bias is important, however, we find that only 21 (34%) studies did this. None of the included studies reports whether the data were missing at random (MAR, MCAR) or not (MNAR). This is a valuable factor when determining whether missing data have contributed to bias in the study and when considering what method to apply to correct for missing data. MNAR assumes that the missingness pattern is dependent on unobserved variables, so it is a potentially greater source of bias.^9^ Only 31% of the studies reported a missing data method, the other studies (69%) may have applied a missing data method without reporting it in the publication. In the absence of any additional missing data methods, a CCA is likely to have been performed. In 2012, it was reported that 81% of epidemiologic studies carry out a CCA.^31^ More recently, it was reported that 70% carry out CCA and 18%, MI.^30^ It has already been recommended that missing data assumptions and the rationale for using a CCA should be reported, since CCA may bias the study estimates as data is often not MCAR.^8^, ^9^ Three of the included studies reported a head‐to‐head comparison: two found that the use of MI compared to CCA did not change the conclusions of the study and one reported difficulty in comparing the methods due to a large quantity of missing data. We recommend that future studies conduct and report sensitivity analyses to clarify the potential impact of methodological choices when addressing missing data in their analyses.^8^

One of the other possible solutions to deal with missing data is MI, which allows the inclusion of the patients who have missing data. It is a method that assumes data are MAR but it can handle data which is MCAR or (under stronger assumptions) MNAR.^15^ MI can improve precision in the study estimate compared to CCA, although its use did not make a substantial impact on the point estimate in the studies which reported using both methods separately.^32^, ^33^, ^34^ Since there was heterogeneity in the databases used, different MI models were applied within the studies to account for the available variables. None of the studies captured in our review applied a statistical method to address systematically missing information. Theoretically, imputation techniques can be applied to the pooled raw data of multiple databases, however, practically this is often not possible due to restrictions on the sharing of data. The use of MI also incorrectly assumes homogeneity of associations amongst (possibly) heterogeneous populations and data sources.^12^ MI can potentially be applied to multi‐level data structures to handle systematic missingness in distributed databases networks (DDNs, e.g., where a central analysis is not possible), by using methods such as MLMI.^12^, ^19^ Secrest et al.^17^ adapted this method and applied it in a DDN with simulated and real‐world data using the UK's Clinical Practice Research Datalink. The authors concluded that this method can be used to reduce bias from systematically missing data by allowing for statistical adjustment if at least one database contains the variables of interest. MLMI and other advanced MI methods have been shown to handle missing data and repair bias in a DDN, however, further research is required for its application in real pharmacoepidemiologic analysis.^16^, ^17^, ^18^, ^19^

**FIGURE 2:**
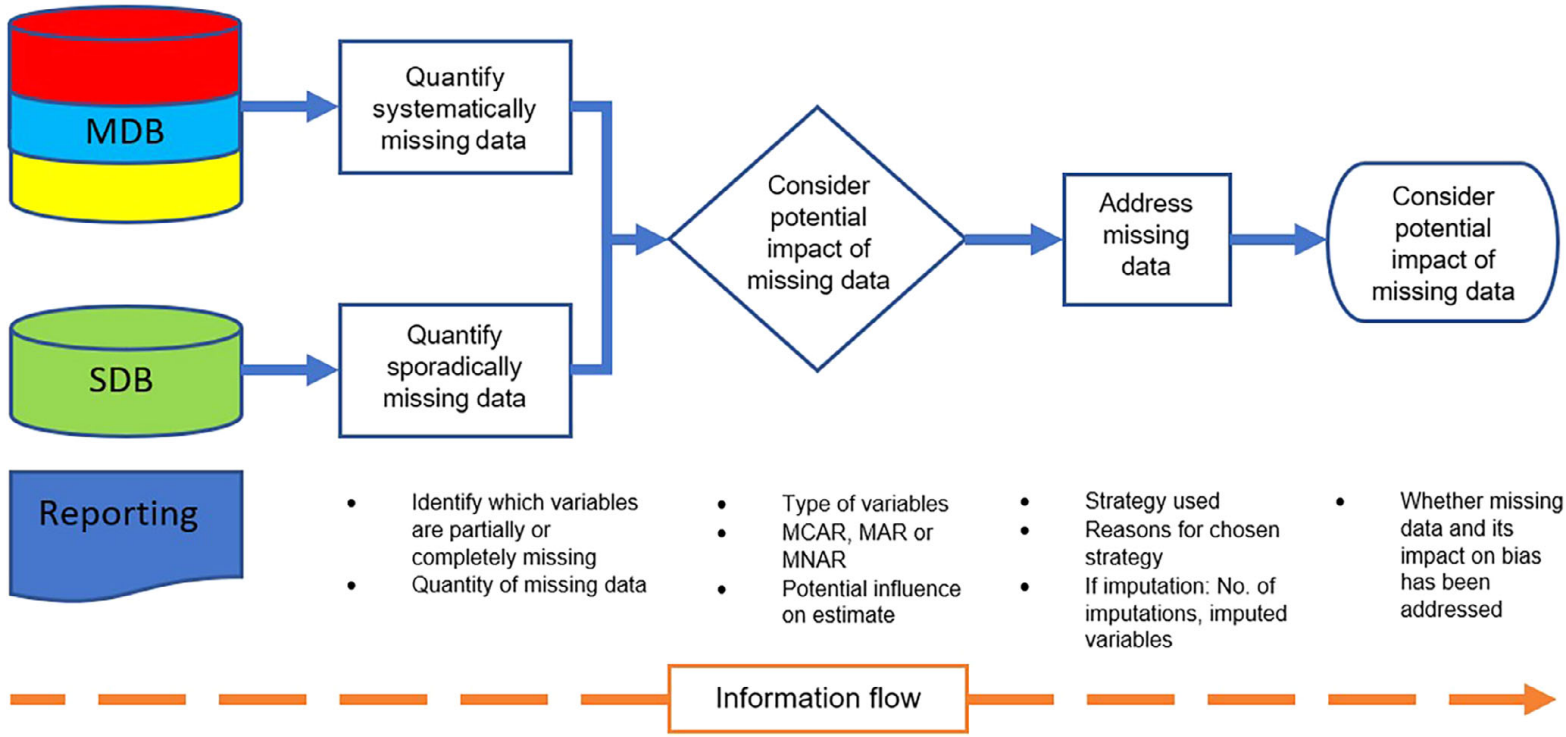
A flow diagram of the suggested process for reporting missing data and the use of missing data methods when publishing a multi‐database pharmacoepidemiologic study. Basic details of missing data and missing data method which should be recorded and at which stage in a multi‐database pharmacoepidemiologic study. MCAR, missing completely at random; MAR, missing at random; MNAR, missing not at random; MDB, multiple databases; SDB, single database [Colour figure can be viewed at wileyonlinelibrary.com]

We believe that researchers, database management teams, reviewers and journal editors should be extra vigilant for data completeness. Compared to single‐database studies, MDBS have the additional complexity of heterogeneity between databases. To give insight into the existence of missing data, we recommend that researchers report the quantity and type of missing data for any variables that are relevant to the analysis or interpretation of results, in line with recommendations in the RECORD‐PE and STROBE statements.^20^, ^21^ In addition, missingness terms (MCAR, MAR, MNAR) should be used to simply demonstrate the potential impact of missing data on the study estimate. MDBS should specifically report whether data are sporadically or systematically missing. This coupled with the use of an appropriate method for addressing missing data will increase overall confidence in the study.^8^, ^21^, ^35^ An overview of the recommended steps in addressing and reporting missing data in MDB pharmacoepidemiologic studies can be found in Figure 2.

In this review, we successfully captured MDB pharmacoepidemiologic studies from multiple regions around the world, expanding on previous work to identify these studies in a systematic search.^1^ To our knowledge, a review of the reporting of missing data and the methods used to address it in MDBS, has not previously taken place. However, there are some potential limitations to our study. First, we were not able to determine with absolute certainty how much data were missing in each study or database, as we were limited to reviewing only what was reported in published studies. Future research could assess the origin and quantity of missing data by directly examining pharmacoepidemiologic databases, particularly before and after data processing, and compare the findings against what is routinely reported for these databases. Second, we might have missed relevant studies due to difficulties in detecting MDBS from systematic searches, as also indicated in similar studies.^1^ For example, studies which use an established database network might not refer to the use of multiple databases in their abstract but instead to the network name. To account for this, we included names of well‐known database networks in our search strategy, which were identified in collaboration with an expert. In addition, we only included publications in English, which could limit the generalisability of our findings beyond Europe and North America.

Multi‐database pharmacoepidemiologic studies are deemed to be essential for regulatory and clinical assessments of drug safety and effectiveness, thus we must increase confidence in the potential that these studies can bring.^36^ Missing data are a persistent problem in EHR, and it is underreported in multi‐database pharmacoepidemiologic studies. The quantity and type of missing data as well as the resulting potential bias, and justification of the method used to address it should be reported.

## CONFLICT OF INTEREST

The authors declare no conflict of interest.

## ETHICS STATEMENT

No ethical approval was required for the study.

## Supporting information


**Data S1**: Supporting information
