# Supplementary material for: A systematic review of how missing data are handled and reported in multi‐database pharmacoepidemiologic studies
Source: Pharmacoepidemiol Drug Saf. 2021 May 7;30(7):819–26. doi: 10.1002/pds.5245 (PMC8252545; doi:10.1002/pds.5245)
Supplement: Supplementary file 1 — Data S1: Supporting information [file PDS-30-819-s001.docx]

Supplementary materials

Supplementary Figure 1. An overview of the location of data sources used in the included multi-databases studies.


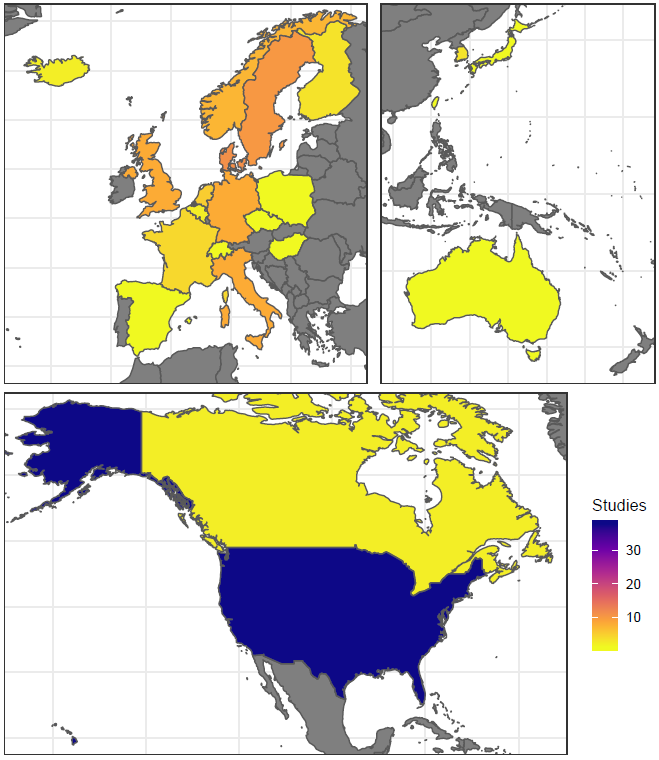


| Supplementary Table 1. The systematic search terms used in PubMed. | | |
| --- | --- | --- |
|  | Search term | Hits |
| #1 | "drug therapy"[MH] OR "drug toxicity"[MH] OR "drug effects" [SH] OR "chemically induced" [SH] OR "complications" [SH] OR "pharmacology" [SH] OR "therapeutic use" [SH] OR "drug therapy" [SH])) OR (drug[TIAB] OR drugs[TIAB] OR prescription[TIAB] OR prescriptions[TIAB] OR medication[TIAB] OR medications[TIAB] OR pharmacotherapy[TIAB] OR "Pharmaceutical Preparations"[Mesh] | 10020581 |
| #2 | "epidemiology"[MH:NoExp] OR "pharmacoepidemiology"[MH] OR "epidemiologic studies"[MH:NoExp] OR "case-control studies"[MH] OR "retrospective studies"[MH] OR "cohort studies"[MH] OR "longitudinal studies"[MH] OR "follow-up studies"[MH] OR "prospective studies"[MH] OR "cross-sectional studies"[MH] OR "epidemiology" [SH:NoExp] OR (pharmacoepidemiologic[TIAB] OR pharmacoepidemiological[TIAB] OR pharmacoepidemiology[TIAB] OR epidemiologic[TIAB] OR epidemiological[TIAB] OR epidemiology[TIAB] OR "case-control"[TIAB] OR "case control"[TIAB] OR cohort[TIAB] OR OR "population based"[TIAB] OR "population-based"[TIAB] OR "nation wide"[TIAB] OR "nation-wide "[TIAB] OR "nationwide"[TIAB] OR "case crossover"[TIAB] OR "case-crossover"[TIAB] OR "case-time-control"[TIAB] OR "case time control"[TIAB] OR "self-controlled case series"[TIAB] OR "self controlled case series"[TIAB] OR "epidemiologic studies" [MESH] OR "pharmacoepidemiology" [MESH] | 3840414 |
| #3 | "registries"[MH] OR "medical records systems, computerized"[MH] OR "databases, factual"[MH:NoExp] OR database[TIAB] OR databases[TIAB] OR "data bases"[TIAB] OR register[TIAB] OR registers[TIAB] OR registry[TIAB] OR registries[TIAB] OR "computerized data"[TIAB] OR "administrative claims"[TIAB] OR "administrative data"[TIAB] OR "claims data"[TIAB] OR combining[TIAB] OR combined[TIAB] OR pooled[TIAB] OR pooling[TIAB] OR "meta-analysis"[TIAB] OR "medical records"[TIAB] OR "patient records"[TIAB] OR "healthcare records"[TIAB] OR "health records"[TIAB] OR registers[TIAB] OR registries[TIAB] OR datasets[TIAB] OR "data sets"[TIAB] OR "data bases "[TIAB] OR "Databases, Factual"[Mesh:NoExp] OR "Databases, Pharmaceutical"[Mesh] | 1809971 |
| #4 | “Risk ratio” OR “Relative risk” OR “Risk difference” OR “rate ratio” OR “Relative rate” OR “Rate difference” OR “Odds ratio” OR “Odds difference” OR “Hazard ratio” OR “Number needed to treat” OR “Number needed to harm” OR “Attributable fraction” OR “Preventable fraction” OR “regression” OR “model*” OR “incidence rate” OR “absolute risk” OR “hazard rate” | 2946345 |
| #5 | "multiple databases" OR "multi-database" OR "multidatabase" OR "multi database" OR "multi-source" OR multi-cent* OR multinational OR "multiple regions" OR multi-country OR "multiple countries" OR multi-cohort OR multi-site OR "multiple sites" OR distributed data* OR "distributed network" OR "database network" OR "research network" OR "drug safety network" OR consortium OR "data partner" OR "common data model" OR "common protocol" OR international OR "geographic areas" OR "cross-country" OR "MDBS" OR provinces[tiab] OR regions[tiab] OR countries[tiab] OR sources[tiab] OR cohorts[tiab] | 1885598 |
| #6 | "clinical trials as topic"[MH] OR "pilot projects"[MH] OR "systematic review"[TIAB]) OR review[title] OR "double-blind"[TW] OR "placebo-controlled"[TW] OR "pilot study"[TW] OR "addresses"[PT] OR "case reports"[PT] OR "comment"[PT] OR "editorial"[PT] OR "guideline"[PT] OR "letter"[PT] OR "published erratum"[PT] OR "randomized controlled trial"[PT] OR "clinical trial"[PT] OR "clinical trial, phase I"[PT] OR "clinical trial, phase II"[PT] OR "clinical trial, phase III"[PT] OR "clinical trial, phase IV"[PT] OR "controlled clinical trial"[PT] | 5297542 |
| #7 | #1 AND #2 AND #3 AND #4 AND #5 | 14730 |
| #8 | #7 NOT #6 | 11835 |
| #9 | #8 Filters: Publication date from 2018/01/01 to 2019/12/31 | 2208 |

| Supplementary Table 2. A list of the included studies | | |
| --- | --- | --- |
| Reference | No. of Databases | Region |
| Babaei, M. et al. Administration of adjuvant chemotherapy for stage II-III colon cancer patients: An European population-based study. Int. J. Cancer 142, 1480–1489 (2018). | 3 | Europe |
| Baker, M. A. et al. Kawasaki disease and 13-valent pneumococcal conjugate vaccination among young children: A self-controlled risk interval and cohort study with null results. PLoS Med. 16, 1–13 (2019). | 6 | North America |
| Bateman, B. T. et al. β-Blocker use in pregnancy and the risk for congenital malformations: An international cohort study. Ann. Intern. Med. 169, 665–673 (2018). | 6 | Combination |
| Belleudi, V. et al. Effectiveness and Safety of Switching Originator and Biosimilar Epoetins in Patients with Chronic Kidney Disease in a Large-Scale Italian Cohort Study. Drug Saf. 42, 1437–1447 (2019). | 4 | Europe |
| Biffi, A. et al. Antidepressants and the risk of arrhythmia in elderly affected by a previous cardiovascular disease: a real-life investigation from Italy. Eur. J. Clin. Pharmacol. 74, 119–129 (2018). | 5 | Europe |
| Cheng, F. et al. Network-based approach to prediction and population-based validation of in silico drug repurposing. Nat. Commun. 9, 1–12 (2018). | 2 | North America |
| Chrischilles, E. A. et al. Prospective surveillance pilot of rivaroxaban safety within the US Food and Drug Administration Sentinel System. Pharmacoepidemiol. Drug Saf. 27, 263–271 (2018). | 4 | North America |
| Czaja, A. S. et al. Electronic health record (EHRs) based postmarketing surveillance of adverse events associated with pediatric off-label medication use: A case study of short‐acting beta‐2 agonists and arrhythmias. Pharmacoepidemiol. Drug Saf. 27, 815–822 (2018). | 2 | North America |
| Daneman, N. et al. Fluoroquinolone use for uncomplicated urinary tract infections in women: A retrospective cohort study. Clin. Microbiol. Infect. (2019) doi:10.1016/j.cmi.2019.10.016. | 6 | North America |
| Dave, C. V. et al. Sodium-glucose cotransporter-2 inhibitors and the risk for severe urinary tract infections. Ann. Intern. Med. 171, 248–256 (2019). | 2 | North America |
| Dave, C. V., Schneeweiss, S. & Patorno, E. Comparative risk of genital infections associated with sodium-glucose co-transporter-2 inhibitors. Diabetes, Obes. Metab. 21, 434–438 (2019). | 2 | North America |
| Dommasch, E. D., Kim, S. C., Lee, M. P. & Gagne, J. J. Risk of Serious Infection in Patients Receiving Systemic Medications for the Treatment of Psoriasis. JAMA Dermatology 155, 1142–1152 (2019). | 2 | North America |
| Donahue, J. G. et al. Inactivated influenza vaccine and spontaneous abortion in the Vaccine Safety Datalink in 2012–13, 2013–14, and 2014–15. Vaccine 37, 6673–6681 (2019). | 6 | North America |
| Dong, Y. H. et al. Use of olmesartan and enteropathy outcomes: a multi-database study. Aliment. Pharmacol. Ther. 47, 792–800 (2018). | 5 | North America |
| Dydensborg Sander, S. et al. Association Between Antibiotics in the First Year of Life and Celiac Disease. Gastroenterology 156, 2217–2229 (2019). | 2 | Europe |
| Eworuke, E., Welch, E. C., Tobenkin, A. & Maro, J. C. Use of FDA’s Sentinel System to Quantify Seizure Risk Immediately Following New Ranolazine Exposure. Drug Saf. 42, 897–906 (2019). | 14 | North America |
| Font, R. et al. Influence of adherence to adjuvant endocrine therapy on disease-free and overall survival: a population-based study in Catalonia, Spain. Breast Cancer Res. Treat. 175, 733–740 (2019). | 2 | Europe |
| Gilsenan, A. et al. Cardiovascular Safety of Prucalopride in Patients with Chronic Constipation: A Multinational Population-Based Cohort Study. Drug Saf. 42, 1179–1190 (2019). | 5 | Europe |
| Given, J. E. et al. Metformin exposure in first trimester of pregnancy and risk of all or specific congenital anomalies: Exploratory case-control study. BMJ 361, 18–20 (2018). | 11 | Combination |
| Glanz, J. M. et al. Association between estimated cumulative vaccine antigen exposure through the first 23 months of life and non–vaccine-targeted infections from 24 through 47 months of age. JAMA - J. Am. Med. Assoc. 319, 906–913 (2018). | 6 | North America |
| Groom, H. C. et al. Uptake and safety of Hepatitis B vaccination during pregnancy: A Vaccine Safety Datalink study. Vaccine 36, 6111–6116 (2018). | 5 | North America |
| Groom, H. C. et al. Uptake and safety of hepatitis A vaccination during pregnancy: A Vaccine Safety Datalink study. Vaccine 37, 6648–6655 (2019). | 5 | North America |
| Hechter, R. C. et al. Vaccine safety in HIV-infected adults within the Vaccine Safety Datalink Project. Vaccine 37, 3296–3302 (2019). | 5 | North America |
| Heerman, W. J. et al. Maternal antibiotic use during pregnancy and childhood obesity at age 5 years. Int. J. Obes. 43, 1202–1209 (2019). | 7 | North America |
| Hoffman, V. et al. Safety study of live, oral human rotavirus vaccine: A cohort study in United States health insurance plans. Hum. Vaccines Immunother. 14, 1782–1790 (2018). | 2 | North America |
| Huybrechts, K. F. et al. Association between methylphenidate and amphetamine use in pregnancy and risk of congenital malformations: A cohort study from the international pregnancy safety study consortium. JAMA Psychiatry 75, 167–175 (2018). | 6 | Combination |
| Hviid, A. et al. Human papillomavirus vaccination of adult women and risk of autoimmune and neurological diseases. J. Intern. Med. 283, 154–165 (2018). | 2 | Europe |
| Jackson, M. L. et al. Safety of repeated doses of tetanus toxoid, reduced diphtheria toxoid, and acellular pertussis vaccine in adults and adolescents. Pharmacoepidemiol. Drug Saf. 27, 921–925 (2018). | 6 | North America |
| Javed, M. A. et al. Impact of intensified chemotherapy in metastatic pancreatic ductal adenocarcinoma (PDAC) in clinical routine in Europe. Pancreatology 19, 97–104 (2019). | 10 | Europe |
| Jin, Y., Kang, E. H., Brill, G., Desai, R. J. & Kim, S. C. Cardiovascular (CV) risk after initiation of abatacept versus TNF inhibitors in rheumatoid arthritis patients with and without baseline CV disease. J. Rheumatol. 45, 1240–1248 (2018). | 2 | North America |
| Kharbanda, E. O. et al. Risk of spontaneous abortion after inadvertent human papillomavirus vaccination in pregnancy. Obstet. Gynecol. 132, 35–44 (2018). | 7 | North America |
| Kim, S. C. et al. Risk of malignancy associated with use of tocilizumab versus other biologics in patients with rheumatoid arthritis: A multi-database cohort study. Semin. Arthritis Rheum. 49, 222–228 (2019). | 3 | North America |
| Kim, S. C. et al. No difference in cardiovascular risk of tocilizumab versus abatacept for rheumatoid arthritis: A multi-database cohort study. Semin. Arthritis Rheum. 48, 399–405 (2018). | 2 | North America |
| Kingwell, E. et al. Multiple sclerosis: Effect of beta interferon treatment on survival. Brain 142, 1324–1333 (2019). | 5 | Combination |
| Krista F. Huybrechts, Chandrasekar Gopalakrishnan, Dorothee B. Bartels, Kristina Zint, Venkatesh K. Gurusamy, Joan Landon, S. S. Safety and effectiveness of dabigatran and other direct oral anticoagulants compared to warfarin in patients with atrial fibrillation. Clin. Pharmacol. Ther. 13, 287–288 (2019). | 2 | North America |
| Kuntz, J., Crane, B., Weinmann, S. & Naleway, A. L. Myocarditis and pericarditis are rare following live viral vaccinations in adults. Vaccine 36, 1524–1527 (2018). | 4 | North America |
| Lai, E. C. C. et al. Comparative safety of NSAIDs for gastrointestinal events in Asia-Pacific populations: A multi-database, international cohort study. Pharmacoepidemiol. Drug Saf. 27, 1223–1230 (2018). | 5 | Combination |
| Li, J. et al. Association of Risk for Venous Thromboembolism with Use of Low-Dose Extended- and Continuous-Cycle Combined Oral Contraceptives: A Safety Study Using the Sentinel Distributed Database. JAMA Intern. Med. 178, 1482–1488 (2018). | 17 | North America |
| Li, X. et al. Apixaban 5 and 2.5 mg twice-daily versus warfarin for stroke prevention in nonvalvular atrial fibrillation patients: Comparative effectiveness and safety evaluated using a propensity-score-matched approach. PLoS One 13, 1–18 (2018). | 4 | North America |
| Lip, G. Y. H. et al. Effectiveness and safety of oral anticoagulants among nonvalvular atrial fibrillation patients: The ARISTOPHANES study. Stroke 49, 2933–2944 (2018). | 5 | North America |
| Lockwood G. Taylor; Genna Panucci; Andrew D. Mosholder; Sengwee Toh; Ting-Ying Huang. Antipsychotic Use and Stroke: A Retrospective Comparative Study in a Non-Elderly Population. J. Clin. Psychiatry 80, (2019). | 13 | North America |
| Malfertheiner, P., Ripellino, C. & Cataldo, N. Severe intestinal malabsorption associated with ACE inhibitor or angiotensin receptor blocker treatment. An observational cohort study in Germany and Italy. Pharmacoepidemiol. Drug Saf. 27, 581–586 (2018). | 6 | Europe |
| Masclee, G. M. C. et al. Risk of acute myocardial infarction during use of individual NSAIDs: A nested case-control study from the SOS project. PLoS One 13, 1–18 (2018). | 6 | Europe |
| McClure, D. L. et al. Similar relative risks of seizures following measles containing vaccination in children born preterm compared to full-term without previous seizures or seizure-related disorders. Vaccine 37, 76–79 (2019). | 7 | North America |
| Meijer, M. et al. Finasteride treatment and male breast cancer: a register-based cohort study in four Nordic countries. Cancer Med. 7, 254–260 (2018). | 4 | Europe |
| Mosholder, A. D. et al. Incidence of heart failure and cardiomyopathy following initiation of medications for attention-deficit/hyperactivity disorder. J. Clin. Psychopharmacol. 38, 505–508 (2018). | 15 | North America |
| Nechanská, B. et al. Neonatal outcomes after fetal exposure to methadone and buprenorphine: national registry studies from the Czech Republic and Norway. Addiction 113, 1286–1294 (2018). | 2 | Europe |
| Persson, F. et al. Dapagliflozin is associated with lower risk of cardiovascular events and all-cause mortality in people with type 2 diabetes (CVD-REAL Nordic) when compared with dipeptidyl peptidase-4 inhibitor therapy: A multinational observational study. Diabetes, Obes. Metab. 20, 344–351 (2018). | 3 | Europe |
| Peterson, E. D., Ashton, V., Chen, Y. W., Wu, B. & Spyropoulos, A. C. Comparative effectiveness, safety, and costs of rivaroxaban and warfarin among morbidly obese patients with atrial fibrillation. Am. Heart J. 212, 113–119 (2019). | 2 | North America |
| Rea, F. et al. Adherence of Elderly Patients with Cardiovascular Disease to Statins and the Risk of Exacerbation of Chronic Obstructive Pulmonary Disease: Evidence from an Italian Real-World Investigation. Drugs and Aging 35, 1099–1108 (2018). | 5 | Europe |
| Ryan, P. B. et al. Comparative effectiveness of canagliflozin, SGLT2 inhibitors and non-SGLT2 inhibitors on the risk of hospitalization for heart failure and amputation in patients with type 2 diabetes mellitus: A real-world meta-analysis of 4 observational databases (OBSER. Diabetes, Obes. Metab. 20, 2585–2597 (2018). | 4 | North America |
| Schink, T. et al. Risk of ischemic stroke and the use of individual non-steroidal anti-inflammatory drugs: A multi-country european database study within the SOS Project. PLoS One 13, 1–14 (2018). | 6 | Europe |
| Simon, T. A. et al. Comparative risk of malignancies and infections in patients with rheumatoid arthritis initiating abatacept versus other biologics: a multi-database real-world study. Arthritis Res. Ther. 21, 1–9 (2019). | 3 | North America |
| Son, N., Kim, B., Chung, S. & Han, S. Korean pharmacovigilance system based on EHR-CDM. Stud. Health Technol. Inform. 264, 1592–1593 (2019). | 9 | East Asia |
| Spence, A. D. et al. Statin use and survival in patients with gastric cancer in two independent population-based cohorts. Pharmacoepidemiol. Drug Saf. 28, 460–470 (2019). | 2 | Europe |
| Sukumaran, L. et al. Infant hospitalizations and mortality after maternal vaccination. Pediatrics 141, (2018). | 5 | North America |
| Tseng, H. F. et al. Pneumococcal conjugate vaccine safety in elderly adults. Open Forum Infect. Dis. 5, 1–8 (2018). | 6 | North America |
| Ueda, P. et al. Sodium glucose cotransporter 2 inhibitors and risk of serious adverse events: nationwide register based cohort study. BMJ 363, k4365 (2018). | 2 | Europe |
| Ungaro, R. C. et al. Stopping 5-aminosalicylates in patients with ulcerative colitis starting biologic therapy does not increase the risk of adverse clinical outcomes: Analysis of two nationwide population-based cohorts. Gut 68, 977–984 (2019). | 2 | Combination |
| Vashisht, R. et al. Association of Hemoglobin A1c Levels With Use of Sulfonylureas, Dipeptidyl Peptidase 4 Inhibitors, and Thiazolidinediones in Patients With Type 2 Diabetes Treated With Metformin: Analysis From the Observational Health Data Sciences and Informatics Initiat. JAMA Netw. open 1, e181755 (2018). | 8 | Combination |
| Wang, H. et al. Insulin analogues use in pregnancy among women with pregestational diabetes mellitus and risk of congenital anomaly: A retrospective population-based cohort study. BMJ Open 8, 1–9 (2018). | 7 | Europe |
| Williams, R. et al. Association between vildagliptin and risk of angioedema, foot ulcers, skin lesions, hepatic toxicity, and serious infections in patients with type 2 diabetes mellitus: A European multidatabase, noninterventional, postauthorization safety study. Endocrinol. Diabetes Metab. 2, e00084 (2019). | 5 | Europe |
